# Phytohormone-Based Regulation of Trichome Development

**DOI:** 10.3389/fpls.2021.734776

**Published:** 2021-09-30

**Authors:** Jinxing Li, Xingxing Wang, Rui Jiang, Boran Dong, Shiyuan Fang, Qing Li, Zongyou Lv, Wansheng Chen

**Affiliations:** ^1^Research and Development Center of Chinese Medicine Resources and Biotechnology, Shanghai University of Traditional Chinese Medicine, Shanghai, China; ^2^Department of Pharmacy, Changzheng Hospital, Second Military Medical University, Shanghai, China

**Keywords:** phytohormones, trichome, gibberellins, endoreduplication, CRISPR/Cas9, sing cell transcriptome

## Abstract

Phytohormones affect plant growth and development. Many phytohormones are involved in the initiation of trichome development, which can help prevent damage from UV radiation and insect bites and produce fragrance, flavors, and compounds used as pharmaceuticals. Phytohormones promote the participation of transcription factors in the initiation of trichome development; for example, the transcription factors HDZIP, bHLH and MYB interact and form transcriptional complexes to regulate trichome development. Jasmonic acid (JA) mediates the progression of the endoreduplication cycle to increase the number of multicellular trichomes or trichome size. Moreover, there is crosstalk between phytohormones, and some phytohormones interact with each other to affect trichome development. Several new techniques, such as the CRISPR-Cas9 system and single-cell transcriptomics, are available for investigating gene function, determining the trajectory of individual trichome cells and elucidating the regulatory network underlying trichome cell lineages. This review discusses recent advances in the modulation of trichome development by phytohormones, emphasizes the differences and similarities between phytohormones initially present in trichomes and provides suggestions for future research.

## Highlights

- Reviewed the phytohormones regulation the development of trichome.
- New technologies may accelerate the study of trichome development.

## Introduction

Trichomes are a type of tissue often localized on plant leaves, buds, and stems and are classified into two types: glandular trichomes and non-glandular trichomes. Glandular trichomes can synthesize and deposit many kinds of secondary metabolites, such as terpenoids, polyketides, phenylpropanoids and alkaloids, which are very useful to humans for the production of commercial products, such as medicines, fragrances, and pigments. Glandular trichomes are multicellular organs and are considered bioengineering reactors, while non-glandular trichomes are usually single cells and are treated as physical barriers to insects or diffusers of ultraviolet light.

Many researchers have focused on trichome development in recent years. Arabidopsis is a model plant genus, and much progress in the understanding of trichome development has been achieved in this genus. The GLABRA 1 (GL1)-GLABRA 3 (GL3)/ENHANCER OF GLABRA 3 (EGL3)-TRANSPARENT TESTA GLABRA 1 (TTG1) trichome development complex has been well studied (Zhao et al., 2008); however, knowledge of the interaction between this complex and phytohormones is limited. Sweet wormwood (*Artemisia annua*), tomato (*Solanum lycopersicum*), and cucumber (*Cucumis sativus*) are the main plant species used as models for studying glandular trichome development (Lv et al., 2017; Du et al., 2020; Xiong et al., 2020), and many studies have indicated that trichome development involves phytohormones (Yan et al., 2017; Chen et al., 2020; Yuan et al., 2021).

Phytohormones are important signaling molecules that play key roles in plant growth and development and determine plant resistance against insects. Both non-glandular trichomes (providing physical defense) and glandular trichomes (providing chemical defense) serve as defenses against herbivorous insects. Many phytohormones are involved in trichome development, such as salicylic acid (SA), jasmonic acid (JA), gibberellins (GAs), abscisic acid (ABA), brassinosteroids (BRs), auxin and cytokinin (CK) (Zhou et al., 2013; Yan et al., 2018; Liu S. et al., 2020; Tan et al., 2020).

Some trichome tissue can produce useful secondary metabolites with commercial value, and trichomes constitute an ideal model system for studying plant cell development and differentiation. Phytohormones are key regulators of trichome development and differentiation, but the understanding of their roles in trichome initiation and development is limited. Nevertheless, recent progress has been made highlighting trichome development, which is summarized in this review.

## When Is the Right Time to Obtain Genes for Trichome Development?

**FIGURE 1:**
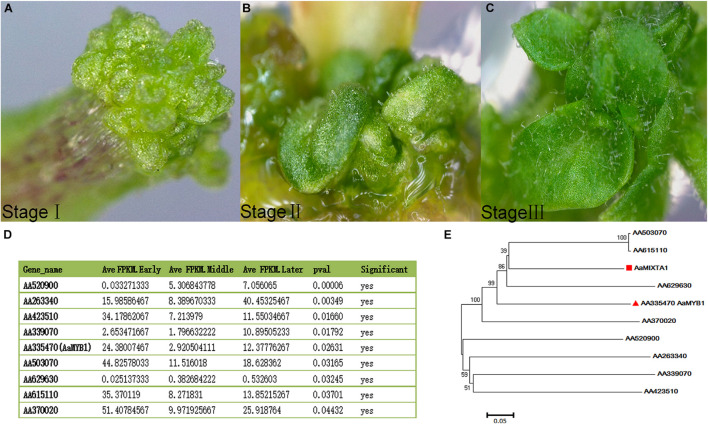
Different developmental stages of *A. annua* calli. **(A)** After 2 weeks of culture on media, calli appeared on the leafstalk (explants). **(B)** Trichomes present on calli after 3 weeks of culture on media. **(C)** Additional trichomes present on calli after 4 weeks of culture on media. **(D)** Nine MYB transcription factors obtained from RNA-seq data across three stages, *p* < 0.05. One of these transcription factors is AaMYB61, which is a positive regulator of trichome development. **(E)** Phylogenetic tree showing that AaMIXTA1 and AA335470 (AaMYB1) are closely related, suggesting that AA503070, AA615110, and AA629630 may play roles in trichome development.

Since model plants have a clear genetic background, ethyl methanesulfonate-induced mutation is a useful method for screening trichome development-related genes in model plants (Payne et al., 2000). However, research on most plant species is lacking; thus, the use of mutagenesis in most plants is limited. The transcriptome is an effective means for screening trichome development-related genes, but choosing the right stage of trichome development is very important. The genes involved in trichome development are dominantly expressed at the primary stage of trichome development; when trichome development is complete, the expression of trichome development-related genes is low or has stopped altogether. Cotton fibers are single-celled trichomes that have differentiated from the ovule epidermis and are considered a model system for studying cell elongation and cell wall biogenesis. The development of fibers can be classified into four stages: the initiation stage [2–5 days post anthesis (DPA)], elongation stage (3–20 DPA), secondary cell wall-deposition stage (16–40 DPA) and maturation stage (40–50 DPA) (Basra and Malik, 1984). Many genes involved in fiber development are expressed predominantly in fibers at 2–20 DPA, such as GhFP1, which is highly expressed in fibers at 9–10 DPA (Liu Z. H. et al., 2020); PAG1, which regulates fiber elongation and is highly expressed in fibers at 15 DPA (Yang et al., 2014); and Gh14-3-3L/e/h, which are expressed mainly in fibers at 3–10 DPA and are positively correlated with the rapid elongation of fibers (Yang et al., 2014). We classified the development of trichome initiation in *A. annua* into three stages: stage I, stage II and stage III (Figures 1A–C). Many genes involved in trichome development in *A. annua* are not expressed or exhibit low expression at the callus stage (stage I); however, at the primary stage of trichome development (stage II), trichome-related genes are highly expressed. At stage III, the expression of genes related to trichome development may be downregulated. Some of these genes have been screened from transcriptome data (Figure 1D), such as AA335470 (AaMYB1), which is a positive regulator of the development of trichomes in *A. annua* (Matias-Hernandez et al., 2017). In addition, new genes with potential roles in the development of trichome development may be found in the phylogenetic tree (Figure 1E), such as AA629630 (closely related to AaMYB1), AA503070 and AA615110 (closely related to AaMIXTA1) (Shi et al., 2018). The raw RNA-seq data are available from the NCBI Sequence Read Archive (accession number PRJNA754709). Therefore, choosing the right stage of trichome development is a key factor for studying trichome development.

## Phytohormones Are Important Regulators of Trichome Development

### Auxin-Based Regulation of Trichome Development

A recent study indicated that auxin is involved in trichome development during tomato and fiber elongation in cotton (Zhang X. et al., 2015; Zhang et al., 2017; Tian and Zhang, 2021). Cotton is not only an important natural-fiber economic crop in the textile industry but also an ideal system for studying genome evolution, polyploidization and cell elongation. Many studies have indicated that auxin plays a positive role in fiber development. A previous study suggested that auxin accumulates in the initial cotton fibers but not in other epidermal cells, indicating that auxin modulates trichome initiation (Zhang et al., 2011). Targeted overexpression of the indole-3-acetic acid (IAA) biosynthesis pathway gene iaaM may increase the IAA content in the epidermis of cotton ovules at the fiber initiation stage. The compound 1-*N*-naphthylphthalamic acid (NPA) can specifically inhibit auxin transport and leads to impaired IAA accumulation and further repression of fiber initiation (Zhang et al., 2011). Repression of the auxin transporter gene *GhPIN* in transgenic cotton has been shown to decrease the expression of the fiber elongation-related genes *GhMYB109*, *GhMYB25*, GhMYB25-like and *GhHD1* and to decrease fiber initiation and elongation (Zhang et al., 2017). NUMEROUS SPINES, which encodes an auxin transporter protein, negatively modulates cucumber fruit spine density (Xie et al., 2018) and may be a target gene that can be used as a marker to improve the quality of cucumber. Taken together, these observations indicate that auxin transporter proteins play important roles in trichome development.

The auxin-based regulation of gene expression depends on AUXIN RESPONSE FACTOR (ARF) and AUXIN/INDOLE-3-ACETIC ACID (Aux/IAA) proteins, both of which bind to promoter regions *via* auxin response elements (AuxREs) (Yamauchi et al., 2019). Aux/IAA proteins have four highly conserved domains, I, II, III and IV, and each determines the functional properties of the protein. Aux/IAA proteins usually act as transcriptional repressors by interacting with ARF proteins (Deng W. et al., 2012). Inhibition of *IAA15* in tomato increases the density of glandular trichome types I and VI and non-glandular trichome type V, indicating that auxin-dependent transcriptional regulation is required for trichome development. Some genes involved in trichome differentiation, such as *GAMYB-like1* and *GAI*, may regulate trichome initiation by interacting with GA signaling (Deng W. et al., 2012). In addition, ARFs can regulate trichome density. In tomato, *ARF3* is expressed mainly in the trichomes. When its expression was inhibited by RNA interference (RNAi), the density of type I, V, and VI trichomes on the leaves decreased, indicating that ARF3 plays a positive role in trichome development (Zhang X. et al., 2015).

**FIGURE 2:**
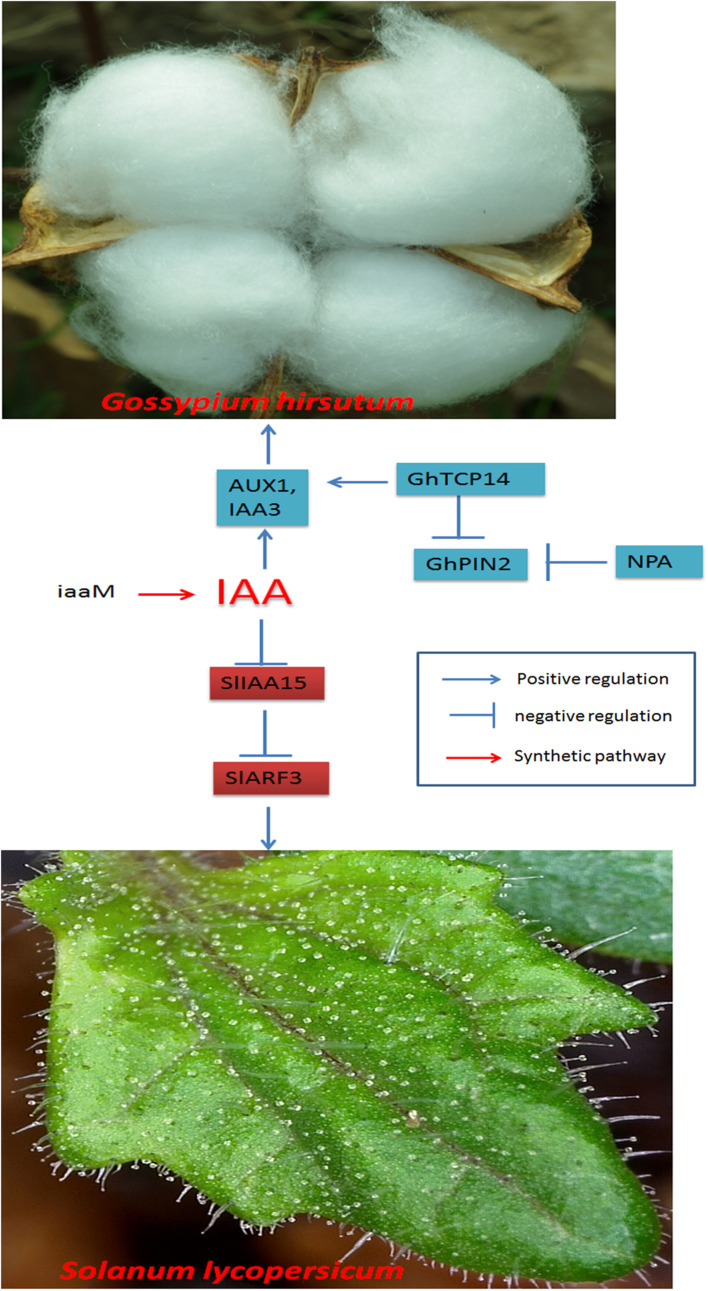
Regulatory networks of cotton fiber and trichome development induced by auxin. NPA negatively regulates the expression of GhPIN2. GhPIN2 is a negative regulator of GhTCP14, and GhTCP14 directly modulates AUX1- or IAA3-promoted fiber initiation in cotton. SlIAA15 is an inhibitor of IAA signaling in tomato and is degraded when the auxin level increases, thus releasing ARFs and activating the transcriptional response.

Auxin increases trichome density by modulating transcription factors. GhTCP14 may bind to the promoters of the auxin efflux carriers *PIN-FORMED 2* (*PIN2*) and *IAA3* and the auxin uptake carrier *AUXIN 1* to increase auxin concentrations, thereby increasing trichome density (Wang et al., 2013; Figure 2).

Previous studies have indicated that abiotic stress-related genes may affect plant growth by decreasing endogenous plant hormone production, thereby increasing trichome density (Johnson et al., 2002; Plett et al., 2010). Overexpression of *Bna.TTG2*, a WRKY transcription factor from *Brassica napus*, increases sensitivity to salt stress and decreases endogenous IAA contents by directly binding to the promoter of the auxin biosynthesis-related genes *TRYPTOPHAN BIOSYNTHESIS 5* and *YUCCA2* (Li et al., 2015). It is suggested that some phytohormones may interact with IAA during trichome development. *PtaMYB186*, an insect resistance gene, inhibits auxin flow and increases ethylene-responsive gene expression, thereby leading to increased foliar trichome density in Populus (Plett et al., 2010). This finding indicates that ethylene may induce trichome development. These genes associated with the abiotic stress response may share the common feature of redirecting the metabolites of auxin to other abiotic stress-related plant hormones, while the production of trichomes may be the result of plant adaptation to the environment.

### Jasmonic Acid-Based Regulation of Trichome Development

Jasmonic acid is a universal phytohormone that regulates trichome development; for example, JA induces trichome initiation in Arabidopsis, *A. annua*, and tomato and trichome elongation in cotton (Traw and Bergelson, 2003; Hu et al., 2016; Yan et al., 2017, 2018; Hua et al., 2021a, b). JA-deficient plants may have reduced numbers of trichomes (Yan et al., 2013); the JA-isoleucine (JA-Ile) receptor may be involved in trichome development (Li et al., 2004). Regulation of the expression of AOS, a JA biosynthesis pathway gene, may increase trichome number (Wu et al., 2008). In the *aos* mutant, mechanical wounding does not lead to an increased number of trichomes. Adding exogenous JA to the *gl3* mutant can cause trichomes to regenerate on Arabidopsis leaves, indicating that JA plays an essential role in trichome development (Yoshida et al., 2009).

In JA signal transduction, JASMONATE-ZIM DOMAIN (JAZ) family proteins serve as JA coreceptors and act as repressors of transcription factors (Kazan and Manners, 2012). Overexpression of JAZ proteins can repress the initiation and elongation of trichomes (Hu et al., 2016); therefore, JAZ proteins may be negative regulators of trichome development. To inhibit trichome initiation, JAZ proteins usually interact together with modulators of trichome development, such as GhMYB25-like in cotton (Hu et al., 2016) and GL3/EGL3, GL1 and TTG1 in Arabidopsis (Qi et al., 2011).

Most trichome regulators reported are transcription factors, such as HDZIP transcription factors, basic helix-loop-helix (bHLH) transcription factors or MYB transcription factors; these include *AtGL2*, *AaHD1*, *AaHD8*, *GhMYB25-like*, and *AtGL3*/*AtEGL3*. HDZIP transcription factors play key roles in trichome development. *AtGL2* in Arabidopsis (Szymanski et al., 1998), *SIWo* in tomato (Yang et al., 2011), *AaHD1* and *AaHD8* in *A. annua* (Yan et al., 2017, 2018), and *CsTril* in cucumber (Du et al., 2020) are HDZIP transcription factors that modulate trichome development.

**FIGURE 3:**
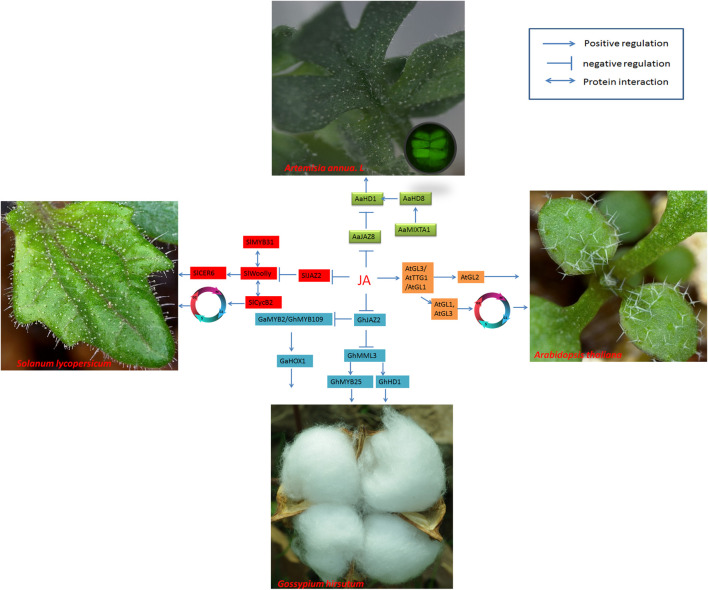
Regulatory networks of cotton fiber and trichome development induced by JA. JAZ proteins are degraded when JA levels increase, releasing ARFs and activating transcription factors such as AaHD1 in *A. annua*, SlWoolly in tomato, and GhMML3 or GhMYB109/GhMYB2 in cotton, thus regulating trichome development or fiber initiation. JA regulates trichome development by affecting the BMW complex to promote the expression of *AtGL2* in Arabidopsis. *AtGL1* and *AtGl3* are involved in cell endoreduplication and induce trichome development. In the development of tomato trichomes, the cyclin protein SlCycB2 interacts with SlWoolly to modulate trichome initiation. SlMYB31 regulates trichome development by modulating lipid loading in trichomes.

The development of trichomes may involve an endoreduplication cycle. Usually, the cell cycle in eukaryotes involves four phases: G1, S, G2, and M. D-type cyclin (CYCD) and cyclin-dependent kinase (CDKA) complexes play important roles in regulating G1→S and G2→M transitions (Ishida et al., 2008; Wang et al., 2020). Trichome initiation occurs at the G1→S and G2→M stages, and regulation of the endoreduplication cycle may be a promising way to modulate multicellular trichomes (Schnittger et al., 2002a, b). Methyl jasmonate (MeJA) may specifically delay the switch from G1→S and prolong the G1 phase (Noir et al., 2013; Wang et al., 2020; Figure 3). Therefore, MeJA may regulate trichome development by modulating endoreduplication.

### Gibberellin-Based Regulation of Trichome Development

The BMW complex is the core element that regulates trichome development in Arabidopsis (Ishida et al., 2008). Trichome initiation is inhibited in GA-deficient mutants, whereas exogenous GA can increase trichome density by upregulating the expression of *GL1*, *TTG1* and *GL3* (Chien and Sussex, 1996; Traw and Bergelson, 2003; Yu et al., 2015). In addition, the mutant *spy-5* (the GA signaling pathway of which is repressed) displays more trichomes than Columbia wild-type Arabidopsis (Col). GL1 may act downstream of the GA signal because the *gl1* mutant is epistatic to the spy mutation (An et al., 2012); results consistent with this observation have been obtained in previous studies. Exogenous application of GA or an increase in the endogenous GA content by overexpression of *GhGA20ox1* may increase endogenous GA levels (especially GA4) in fibers and ovules and promote cotton fiber initiation and elongation (Xiao et al., 2010). These observations show that GA is the main phytohormone involved in trichome development.

GA regulates trichome development mainly by activating the expression of transcription factors that are involved in trichome development. *ZFP5*, a recently discovered C2H2 zinc finger protein involved in GA signaling, plays an important role in trichome development. The number of trichomes on sepals, caulines, and paraclades has been found to be reduced in *zfp5* mutants compared with wild-type plants, while in ZFP5-overexpression plants, the trichome number is increased. *zfp5* acts upstream of *GL1*, *GL3*, *GIS*, *GIS2*, and *ZFP8* and directly targets ZFP8 to modulate epidermal cell differentiation (Zhou et al., 2011). In addition, ZFP8 and GIS2 are targets of GIS3 (Sun et al., 2015). The mRNA level of *ZFP5* is regulated by ZFP6, which modulates trichome development by integrating GA and CK signaling (Zhou et al., 2013). However, the relationship between GIS3 and ZFP6 is poorly understood. Genetic information has suggested that the R3 MYB transcription factor *TCL1* may act downstream of the BMW complex, while the mRNA level of TCL1 is not affected by the BMW complex (Zhang et al., 2018). Thus, posttranscriptional regulation of TCL1 may occur.

**FIGURE 4:**
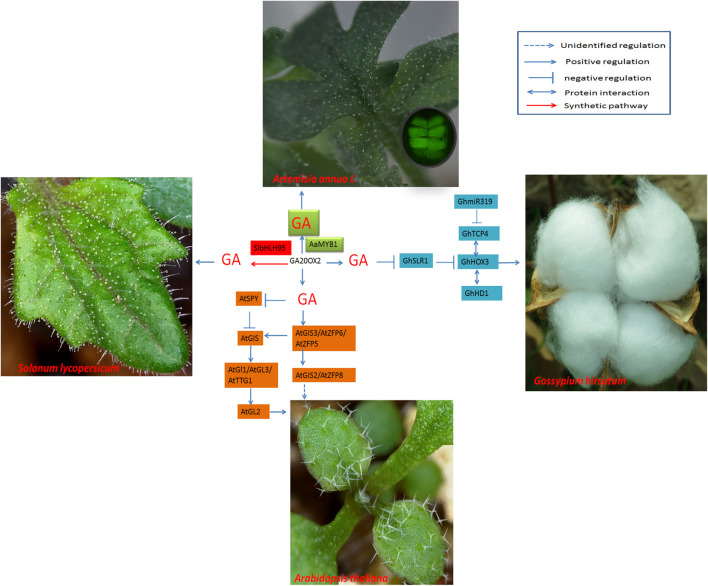
Regulatory networks of cotton fiber and trichome development induced by GA. GA20ox2 is the key enzyme that affects GA content and promotes trichome development. Modulation of GA20ox2 by SlbHLH95 in tomato or by AaMYB1 in *A. annua* affects trichome initiation.

DELLA proteins are inhibitors of GA signal transduction. DELLAs bind to *GhHOX3* and inhibit its binding activity to target genes or its transcriptional activation activity. GA triggers the degradation of DELLAs, which causes the release of GhHOX3; GhHOX3 in turn interacts with other proteins (such as GhHD1) and activates downstream genes to promote cotton fiber elongation (Shan et al., 2014; Figure 4).

### Ethylene-Based Regulation of Trichome Development

Ethylene is involved in trichome development (Zhang Y. et al., 2021). Ethylene stimulates endoreduplication of epidermal cells and increases the rate of cell division, resulting in an abundance of trichomes. Ethylene also alters cell division polarity, leading to the production of branched trichomes (Kazama et al., 2004). *Lotus japonicus lot1* mutant seedlings are insensitive to ethylene treatment and exhibit a phenotype that has fewer trichomes than wild-type seedlings (Ooki et al., 2005). Thus, there may be a direct relationship between ethylene and trichome development.

The trichomes of the Arabidopsis ethylene receptor mutant *etr2-3* are abnormally shaped and unbranched. *ETR2* affects the microtubule cytoskeleton, stabilizing or depolymerizing it (Plett et al., 2009). Microtubule cytoskeleton proteins are involved in the regulation of trichome cell expansion, trichome branch number and the elongation of trichome branches (Tian et al., 2015; Chen et al., 2016; Chang et al., 2019). Therefore, the ethylene signaling pathway may affect trichome development *via* microtubule cytoskeleton proteins. Inhibition of *GbPDF1*, a fiber initiation- and early elongation-related gene, decreases the mRNA levels of several genes related to ethylene, revealing a direct relationship between ethylene and trichome development (Deng F. et al., 2012).

**FIGURE 5:**
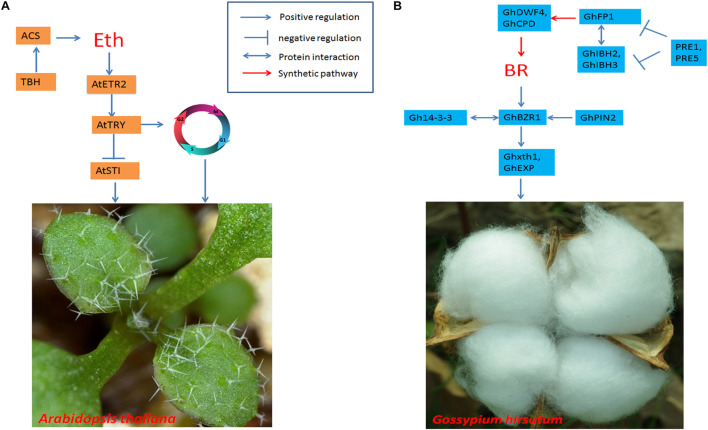
Regulatory networks of trichome development induced by ethylene and for cotton fiber regulated by BRs. **(A)** Ethylene regulates the expression of AaTRY, which is a negative regulator of trichome development in Arabidopsis. It also affects trichome initiation *via* its involvement in cell endoreduplication. **(B)** BRs activate GhBZR1, which is an important transcription factor that regulates BR-responsive gene expression. GhBZR1 promotes the expression of Ghxth1 and GhEXP to modulate fiber initiation in cotton.

Further investigation of the relationship between ethylene and trichome development has revealed that the *TINY BRANCHED HAIR* (*TBH*) gene is preferentially expressed in multicellular trichomes of cucumber. *TBH* can restore the trichome phenotype of *tbh* mutants. Moreover, exogenous ethylene can rescue the trichome defects of *tbh* mutants. *TBH* may directly bind to the promoter of the 1-aminocyclopropane-1-carboxylate synthase (ACS) gene to modulate the accumulation of ethylene, thereby affecting trichome development (Zhang Y. et al., 2021; Figure 5A).

### Brassinosteroids-Based Regulation of Trichome Development

Cotton fibers are single-celled trichomes derived from the epidermal cells of seeds, and BRs are related to the elongation of these fibers, with genes of the BR biosynthesis pathway being upregulated during the initial fiber and elongation stages (Yang et al., 2014). Exogenous BRs promote fiber elongation from cotton ovules, while treatment with brassinazole, a BR biosynthesis inhibitor, can inhibit fiber elongation (Sun et al., 2005). The BR biosynthesis pathway gene PAG1 is a homolog of Arabidopsis *CYP734A1*/BAS1 and plays an important role in the inactivation of BRs through C-26 hydroxylation. PAG1 affects the endogenous biological activation of BRs, thereby affecting ethylene signal transduction by mediating very long-chain fatty acid (VLCFA) levels and regulating fiber elongation (Yang et al., 2014). Another BR biosynthesis pathway-related gene, *DET2*, which encodes a steroid 5d-reductase, may participate in a rate-limiting step in BR biosynthesis. Altering the metabolic flux of the BR biosynthesis pathway by overexpression of GhDET2 affects fiber initiation and fiber elongation. Inhibition of GhDET2 by an antisense method has been shown to reduce fiber initiation and fiber elongation, while the addition of finasteride, a steroid 5a-reductase inhibitor, represses fiber elongation (Luo et al., 2007).

The BR biosynthesis pathway has been thoroughly characterized (Huaxun, 2011). Therefore, the regulation of BR biosynthesis is a promising way to modulate the development of cotton fibers. Liu (Liu Z. H. et al., 2020) overexpressed the bHLH transcription factor *GhFP1* in Arabidopsis to increase trichome length. Moreover, the overexpression of *GhFP1* in cotton can promote fiber elongation. GhFP1 can directly bind to the promoters of *GhCPD* and *GhDWF4*, both of which are BR biosynthesis pathway genes; in turn, this binding positively regulates BR biosynthesis and increases fiber elongation (Figure 5B).

14-3-3 acidic regulatory proteins are highly conserved among eukaryotes. These proteins usually form dimers and alter the relocation, activity, phosphorylation state and stability of their target proteins (Zhang et al., 2010). Gh14-3-3a, Gh14-3-3e, and Gh14-3-3L in cotton may function synergistically in BR signal transduction by altering the nuclear export of BZR1 and promoting the atypical longitudinal growth of cells, thus promoting cotton fiber elongation (Zhang et al., 2010). Overexpression of Gh14-3-3L in cotton can promote fiber elongation and increase mature fiber length, whereas inhibition of the expression of Gh14-3-3L, Gh14-3-3e, and Gh14-3-3 leads to short cotton fibers. 2,4-Epibrassinolide treatment can rescue the short-fiber phenotype in cotton plants in which 14-3-3 proteins are silenced *via* RNAi. Gh14-3-3 interacts with GhBZR1 *via* protein–protein interactions to modulate BR signaling, ultimately affecting fiber initiation and elongation (Zhou et al., 2015).

### Cytokinin-Based Regulation of Trichome Development

Previous studies have indicated that CKs may induce trichome development (Greenboim-Wainberg et al., 2005). Overexpression of the CK biosynthesis-related gene isopentenyl transferase driven by the carpel-specific CRABS CLAW promoter of Arabidopsis produces numerous ectopic trichomes in hairless carpels (Greenboim-Wainberg et al., 2005). ARR is a type-B response regulator that acts as a transcription factor in CK signaling (Kim and Hwang, 2012). Specifically, ARR1, ARR2, ARR10 and ARR12 can interact with SPLs (SPL2, SPL9 and SPL10) to modulate shoot regenerative capacity (Zhang T. et al., 2015). SPL2, SPL9, and SPL10 participate in trichome development by interacting with ARR2 (Shikata et al., 2009; Zhang T. et al., 2015). SPL9 is the direct target of miR156 and regulates trichome initiation by binding to the promoters of TRY and TCL1 (Yu et al., 2010). Mutation of the ARR2 gene in rice leads to a substantial decrease in trichome initiation and elongation in grain hulls and decreased expression of GL3A (a homologous gene of Arabidopsis GL3) (Worthen et al., 2019). 6-Benzylaminopurine (6-BA) is a CK that can strongly increase the transcript levels of ARR5, leading to significant increases in the expression of *GIS2*, *GL1*, and *GIS3* (Gan et al., 2007; Sun et al., 2015).

In a study, the promoter of GIS3 was fused to β-glucuronidase (GUS), after which pGIS3:GUS transgenic plants were generated and treated with 6-BA. Histochemical staining of GUS activity indicated that the CK signal could induce GIS3 expression at the protein level (Sun et al., 2015). GIS3 directly targets GIS2, one of the C2H2 transcription factors that respond to CKs, and regulates trichome development. However, the factors downstream of GIS2 remain unclear. In addition to the C2H2 zinc finger proteins ZFP5, ZFP6, ZFP8, GIS, GIS2 and GIS3, which regulate trichome development (Gan et al., 2007; Zhou et al., 2011, 2013; Sun et al., 2015), another member of the C2H2 zinc finger protein in tomato can regulate trichome development and interact with CKs *via* an unknown mechanism (Chang et al., 2018). In addition, the 26S proteasome can regulate trichome development by modulating endoreduplication (Sako et al., 2010). RPN1a, a subunit of the 26S proteasome, has been shown to be repressed by CK and to inhibit trichome development by downregulating the expression of *ZFP6*, *ZFP5*, *GIS*, *GL1*, *GL2*, *GL3*, *TTG1* and *MYB23* (Yu et al., 2015). GL3 and RHL1 may be involved in the positive modulation of endocycle progression in trichomes (Sako et al., 2010), and *RPN1a* may promote the interaction between CK and endoreduplication to regulate trichome development.

**FIGURE 6:**
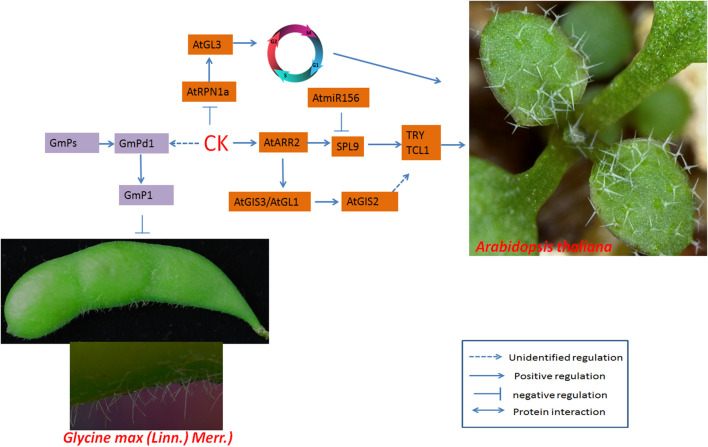
Regulatory networks of cotton fiber and trichome development induced by CKs. The CK receptor AtARR2 interacts with AaSPL9, which is involved in trichome development in Arabidopsis. In soybean, the expression of GmPd1 is induced by CKs, and GmPd1 binds directly to the promoter of GmP1 (which encodes a lipid transporter) and promotes trichome development. ARR, nuclear-localized B-type ARABIDOPSIS RESPONSE REGULATOR.

The interaction between GA and CK affects the expression of *Pd1* (a homolog of soybean *GL2*), thereby activating the expression of lipid transfer protein *P1* (Figure 6), which is localized to the cytoplasm and cell membrane; the transferred lipids affect trichome development (Liu Z. H. et al., 2020).

### Salicylic Acid-Based Regulation of Trichome Development

Salicylic acid plays an important role in resistance to pathogens; it also regulates trichome density. SA at a concentration of 0.1 or 1 mM can reduce trichome density, while different concentrations of SA have no differential effects on trichome density; different varieties of Arabidopsis yield similar results (Traw and Bergelson, 2003). SA can interact with JA to exert various functions (Liu et al., 2016). JA is a positive regulator of trichome size and trichome density (Maes et al., 2011; Hua et al., 2021a, b), and SA can inhibit JA signaling by degrading ORA59 in Arabidopsis. In trichome development, SA negatively regulates trichome density and constitutively weakens the effect of JA, indicating that crosstalk occurs between the SA- and JA-dependent trichome development pathways (Traw and Bergelson, 2003).

Constitutive pathogen response 5 (cpr5) may be involved in trichome development (Bowling et al., 1997; Kirik et al., 2001; Aki et al., 2007). Compared with the wild type, the cpr5 mutant has fewer trichome branches, and the number and size of the trichomes are much smaller.

The DNA content in the trichomes highly significantly differs between the wild type (32C) and cpr5 mutant (8C), indicating that endoreduplication cycles stop prematurely in the mutant (Kirik et al., 2001). In *cpr5* mutants, the content of SA is 20-fold higher than that of Col-0 (Aki et al., 2007). The high concentrations of SA inhibit trichome development (Traw and Bergelson, 2003), leading to fewer trichomes in the *cpr5* mutant. Interactions also occur between SA and GAs. GAs have been shown to increase the number of trichomes by 72.0%; however, this increase was reduced to 29.6% when SA was also added (Traw and Bergelson, 2003). The mechanism underlying these interaction effects is still unknown.

### Phytohormone-Based Regulation of Trichome Size

Stress is an external condition that can affect plant growth and development. Stress can activate the SA, ABA and JA biosynthesis pathways, generating phytohormone responses to external conditions. JA increases the size of trichomes in both *A. annua* and Arabidopsis, GA3 increases the size of trichomes only in *A. annua*, and 6-BA increases the size of trichomes in Arabidopsis (Maes et al., 2008, 2011). Enhancing BR biosynthesis or increasing endogenous GA content can positively regulate fiber elongation in cotton (Xiao et al., 2010; Liu Z. H. et al., 2020). Drought can increase ABA signal transduction, which participates in the initial formation of trichomes by inducing the expression of *MYB41*, which modulates cuticle loading on the trichome surface (Cominelli et al., 2010). However, ABA can antagonize JA, which regulates trichome size (Maes et al., 2011); thus, trichomes decrease in size in response to ABA (Yadav et al., 2014).

Endoreduplication has an important role in modulating plant organ or tissue size (Peng et al., 2015; Li et al., 2020). Most studies on this topic have indicated that cyclin proteins are downstream target genes involved in organ size (Breuer et al., 2009, 2012). The trihelix transcription factor GT2-like 1 (GTL1) can directly inhibit the expression of *CDH1*/*FZR*/CCS52, which is an activator of the APC/C complex/loop body, preventing the development of the endothelial cycle and ploidy-dependent cell growth (Breuer et al., 2012). Furthermore, *GTL1* participates in SA metabolism and signaling by directly binding to the promoter region or genomic region (Völz et al., 2018). SA can antagonize JA during trichome development (Traw and Bergelson, 2003), so SA can enhance the negative effect of *GTL1* on trichome size in Arabidopsis and lead to a decrease in trichome size. However, SA increases the size of glandular trichomes in *A. annua* (Kumari et al., 2018). Thus, models of trichome development can differ between glandular trichomes and non-glandular trichomes.

Phytohormones can affect the expression of the MEP pathway genes 1-deoxy-D-xylulose 5-phosphate synthase and 1-deoxy-D-xylulose-5-phosphate reductoisomerase (Paetzold et al., 2010; Xing et al., 2010), which regulate the contents of terpenoids such as monoterpenes and sesquiterpenes, leading to feedback regulation of trichome development. The monoterpene β-ocimene acts as a phytohormone and can induce trichome development and increase trichome size (Xiao et al., 2020).

## Concluding Remarks

In this review, we discuss the plant hormones that regulate the development of trichomes (Table 1), describe the potential mechanisms affecting trichome density, and emphasize that transcription factors play an important role in the initial development of trichomes. In addition, the mechanisms of development differ between unicellular and multicellular trichomes. Elucidating this difference may help efforts to convert unicellular trichomes to multicellular trichomes and thereby increase the production of secondary metabolites.

**TABLE 1:** Summary of phytohormones-mediated regulators modulation of trichome development.

Hormone	Gene	Targets	Features	References
Auxin	GhiaaM	–	Increases IAA content and promotes cotton fiber initiation	Zhang et al., 2011
	GhPIN	GhMYB109, GhMYB25, GhMYB25-like, GhHD1	Positively regulates fiber elongation	Zhang et al., 2017
	CsNS	CsARF, CsAux/IAA proteins	Negatively regulates fruit spine development	Xie et al., 2018
	SlIAA15	–	Inhibition of trichome types I, VI and V	Deng W. et al., 2012
	SlARF3	–	Increase trichome density of type I, V and VI	Zhang X. et al., 2015
	GhTCP14	GhPIN2, GhIAA3, and GhAUX1	Elongation of trichomes and density, dwarf plant	Wang et al., 2013
	Bna.TTG2	AtTRY5 and AtYUC2	Increase trichome density by affecting auxin accumulation	Li et al., 2015
JA	COI1	–	Affect JA accumulation	Li et al., 2004
	GmAOS	–	Affect JA accumulation and increase trichome density	Wu et al., 2008
	GhJAZ2,SlJAZ2/4,AaJAZ4, AtJAZ1/2/8/10	GhMYB25-like, GhGL1,GhMYC2, GhWD40,GhJI1,SlHD8, AaHD1,Slwo/SlMYC1, AtGL3/EGL3,AtGL1,AtTTG1	Inhibition of fiber or trichome initiation	Qi et al., 2011; Hu et al., 2016; Yan et al., 2017; Hua et al., 2021a, b
GA	GhGA20ox1	–	Enhance endogenous GA levels, promote cotton fiber initiation and elongation	Xiao et al., 2010
	AtSPY	–	Causes GA deficiency and reduce trichome number	Jacobsen and Olszewski, 1993
	AtGL1	AtGL3/EGL3, AtTTG1	Increase trichome density	An et al., 2011
	AtZFP5	AtGL1, AtGL3, AtGIS, AtGIS2, At ZFP8	Regulation of trichome initiation	Zhou et al., 2011
	AtZFP6	AtGIS, AtGIS2, AtZFP8, AtZFP5,AtGL1, AtGL3.	Regulation of trichome initiation	Zhou et al., 2013
	GhSLR1	GhHOX3–GhHD1	Modulation of GA signal to regulate fiber cell elongation.	Shan et al., 2014
Ethylene	ETR2	–	Affects the microtubule cytoskeleton and trichome branch number	Plett et al., 2009
	GhPDF1	GhHDZIP2ATATHB2	Steady biosynthesis of ethylene	Deng F. et al., 2012
	CsTBH	1-Aminocyclopropane-1-Carboxylate Synthase	Accumulation of ethylene	Zhang Y. et al., 2021
BR	GhPAG1	–	Accumulation of endogenous BRs	Yang et al., 2014
	GhDET2	–	Participate in BR biosynthesis and regulation of fiber initiation and elongation	Luo et al., 2007
	GhFP1	GhCPD and GhDWF4,	Regulation of BR biosynthesis and promotion of trichome development	Liu Z. H. et al., 2020
	Gh14-3-3a/e/L	GhBZR1	Regulation of fiber initiation and elongation by modulating brassinosteroid signaling	Zhou et al., 2015
CK	AtARR1/2/10/12	AtSPL2, AtSPL9, AtSPL10	Regulation of trichome	Yu et al., 2010; Zhang T. et al., 2015
	OsARR2	OsGL3A	Positively regulator of trichome development	Worthen et al., 2019
	AtGIS3	AtGIS2	Positively regulator of trichome development	Gan et al., 2007
	AtRPN1a	AtZFP6,AtZFP5, AtGIS, AtGL1, AtGL2, AtGL3, AtTTG1,At MYB23	A negative regulator of trichome development	Yu et al., 2015
	GmPd1	GmP1	Response to CK	Liu S. et al., 2020
SA	Atcpr5	JA,GA	Regulation of trichome development	An et al., 2011; Aki et al., 2007

*At, Arabidopsis thaliana; Aa, Artemisia annuas; Os, Oryza sativa; Ba, Brassica napus; Gm, Glycine max (L) Merr; Cs, Cucumis sativus; Gh, Gossypium hirsutum.*

## Future Perspectives

### Target Modulation of Multicellular Trichomes by Endoreduplication

Fragrances, pigments, and medicines have economic value, and many of these are produced in the glandular trichomes of plants. In *A. annua*, there are two kinds of trichomes: 10-celled glandular trichomes and non-glandular trichomes (Duke and Paul, 1993). Artemisinin is synthesized mainly in glandular trichome cells, although it has been found that non-glandular trichomes produce some artemisinin (Duke and Paul, 1993; Judd et al., 2019). Increasing the number of multicellular trichomes may be a promising method to increase artemisinin content. Cyclin proteins involved in the process of the endoreduplication cycle have the potential to reshape single-celled trichomes into multicellular trichomes, and promising methods to leverage this capacity have the potential to achieve ideal production capacity, offering economic value (Ishida et al., 2008).

CYCD proteins can induce cellular growth and trigger the reprogramming of mitosis or endoreduplication, leading to the production of multicellular trichomes. The cells of plants with a mutation in CYCD3;1 cannot enter S phase (Perazza et al., 1999). Overexpression of CYCD3;1 can shorten the G1 phase and promote the entry of S phase and induce mitosis, resulting in multicellular trichomes (Schnittger et al., 2002a; Menges et al., 2006). The function of *CYCD3* (*CYCD3;1-3*) is dependent on CKs, as evidenced by the impairment of the *cycd3;1-3* mutant in the absence of CKs (Dewitte et al., 2007). These observations indicate that CKs can affect multicellular trichomes through endoreduplication.

CYCB also modulates multicellular trichomes. Ectopic expression of *CYCLIN B1;2* in Arabidopsis can trigger mitotic divisions and lead to multicellular trichomes (Schnittger et al., 2002b). Dominant-negative cyclin-dependent kinase (CDK) or CDK inhibitor proteins (ICK/KRPs) can affect the endoreduplication of trichomes. Misexpression of ICK1/KRP1 inhibits cell differentiation and triggers cell death (Schnittger et al., 2003). Overexpression of ICK1/KRP1 has a positive role in the regulation of multicellular trichomes. SIAMESE (SIM) is a CDK inhibitor involved in endoreplication in Arabidopsis. *sim* mutants exhibit promoted transition from S phase to G2 phase and have an increased number of multicellular trichomes (Churchman et al., 2006). Moreover, overexpression of *SIM* can inhibit CDK complexes and repress the transition from the S phase to G2 phase (Wang et al., 2020).

### CRISPR-Cas9-Mediated Gene Editing for Increasing Trichome Numbers

The bacterial clustered, regularly interspaced, short palindromic repeat (CRISPR)/CRISPR-associated 9 (Cas9) system stems from *Streptococcus pyogenes* and involves specific binding to a DNA sequence (20 bp) guided by an engineered single-guide RNA through RNA–DNA interactions (Li et al., 2013; Nekrasov et al., 2013). CRISPR-Cas9 is a tool for base editing involving nucleotide substitution. It is widely used in genome editing and is characterized by low cost and ease of targeting genetic manipulations. This method can be used to improve the economically important traits of crop species; examples of such use include editing the MILDEW-RESISTANCE LOCUS gene to obtain powdery mildew-resistant wheat (Wang et al., 2014); knocking out *GW2*, *GW5*, and *TGW6* to increase the grain weight of rice (Xu et al., 2016); and targeting the waxy allele to accelerate the breeding process of corn (Gao et al., 2020).

**FIGURE 7:**
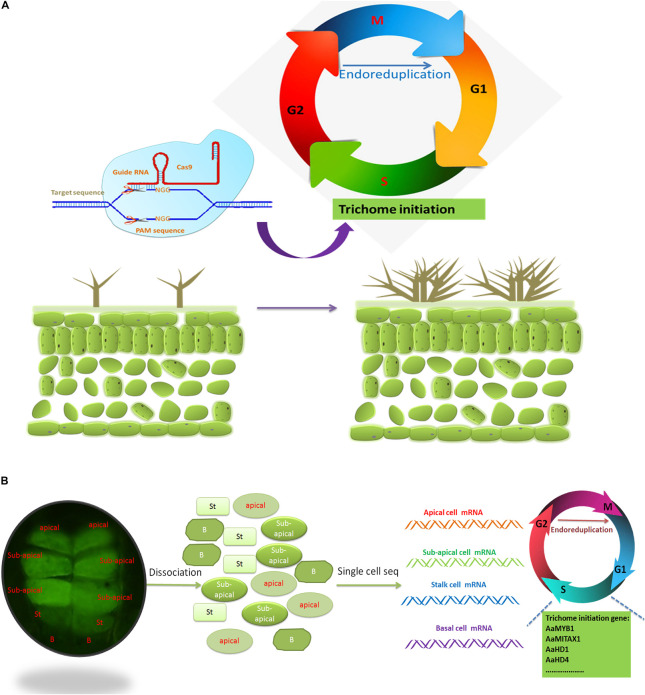
CRISPR-Cas9 and single-cell sequencing may help reveal the development of trichomes. **(A)** CRISPR-Cas9 may be a promising technique for producing multiple trichomes by editing the redundant cyclin genes involved in trichome development. G1, gap 1; G2, gap 2; M, mitosis; S, synthesis. **(B)** Single-cell sequencing can be used to elucidate the mechanism underlying trichome development. Autofluorescence of glandular trichomes on the buds of *A. annua*, as revealed *via* a fluorescent isothiocyanate (FITC) filter (λex = 480 nm; λem = 535 nm). Each trichome of *A. annua* is composed of ten cells: one pair of apical cells, two pairs of subapical cells, one pair of stalk cells and one pair of base cells. The base cells do not autofluorescence. Advanced single-cell mRNA sequencing (mRNA-seq) can be used to restore the developmental process of each cell of glandular trichomes, and the main genes involved in trichome development encode cyclins. St, stalk cell; B, base cell.

There are many negative regulators during trichome development, such as *CPC*, *ETC1*, *ETC1*, *ETC3*, *MYBL2*, *myb82*, *TCL1*, *TCL2*, and *TRY* (Wada et al., 1997; Szymanski and Marks, 1998; Sawa, 2002; Kirik et al., 2004a, b; Wang et al., 2007; Tominaga et al., 2008; Gan et al., 2011; Liang et al., 2014). Simultaneous mutations in *try*/*cpc*/*etc1* lead to a dozenfold increase in the number of trichomes (Wang et al., 2008). Therefore, simultaneous editing of negative regulators of trichomes may be a promising method for obtaining trichome-rich plants (Figure 7A).

### Single-Cell RNA Sequencing Reveals Spatiotemporal Trichome Development

In multicellular organisms, different types of cells with the same genome have unique developmental programs and different functions. It is therefore very important to reveal how genes reach the right state at the right place and at the right time. Although many RNA-seq techniques have been used to study the development of glandular trichomes (Li et al., 2020; Liao et al., 2020), progress in understanding glandular trichome development has been very slow. Since many glandular trichomes are multicellular, the function of each cell is different, resulting in different levels of mRNA in each cell. Detection of mRNA of single-cell or glandular trichomes may be a promising method to study trichome development. Single-cell transcriptomics is a promising method applicable for cells with high or low expression to study new cell types and improve the understanding of the relationship between time and space in real time (Figure 7B).

Glandular trichomes are multicellular trichomes, and each cell can have different functions in the biosynthesis or deposition of secondary metabolites (Olsson et al., 2009). The glandular trichomes of *A. annua* comprise 10 cells: one pair of apical cells, two pairs of subapical cells, one pair of stalk cells and one pair of base cells. The four pairs of cells have different cellular contents according to slicing data (Duke et al., 1994). Therefore, the five classes of cells can exhibit heterogeneity and coregulation of artemisinin biosynthesis. What are the relationships among the five types of cells in regulating the synthesis of artemisinin? What role does each cell play in artemisinin biosynthesis? Single-cell transcriptomics can be a good way to reveal the developmental trajectory of each type of trichome cell and study dynamically expressed genes. The single-cell sequencing is based on protoplast sequencing or transposase-accessible chromatin sequencing (ATAC-seq). The protoplast method was derived from cell wall digestion and dissociation by cellulase and pectinase (Shen et al., 2014). Trichome cells are resistant to cell wall digestion due to the wax and cutin on their surface. Enzymatic digestion procedures can affect gene expression and therefore may not reveal the true level of gene expression (Denyer et al., 2019). Single-cell ATAC-seq can measure the dynamic accessibility of chromatin to gene expression (Farmer et al., 2021; Zhang T. Q. et al., 2021); thus, it can be used to avoid obtaining protoplasts and for single-cell sequencing of glandular trichomes.

Therefore, single-cell sequencing can accurately reveal the function of each cell of glandular trichomes in real time, paving the way for regulating the number of glandular trichomes or for the artificial targeted synthesis of glandular trichomes, thereby leading to increased numbers of economically valuable products.

## Author Contributions

ZL and WC conceived and designed the entire research plans. JL, XW, and SF performed most of the work. QL provided technical assistance. JL and XW wrote the manuscript. WC, RJ, and ZL helped with the organization and editing. All authors contributed to the article and approved the submitted version.

## Conflict of Interest

The authors declare that the research was conducted in the absence of any commercial or financial relationships that could be construed as a potential conflict of interest.

## Publisher’s Note

All claims expressed in this article are solely those of the authors and do not necessarily represent those of their affiliated organizations, or those of the publisher, the editors and the reviewers. Any product that may be evaluated in this article, or claim that may be made by its manufacturer, is not guaranteed or endorsed by the publisher.
